# QuickStats

**Published:** 2014-01-03

**Authors:** FROM THE NATIONAL CENTER FOR HEALTH STATISTICS

**Figure:**
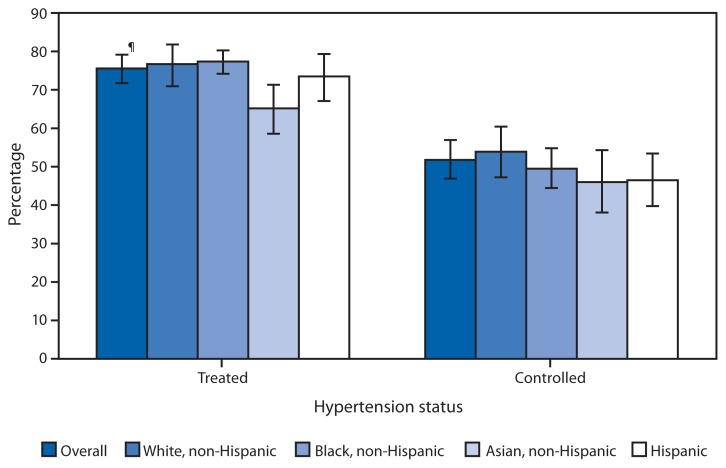
Percentage of Adults Aged ≥18 Years with Hypertension Reporting Treatment* and Control^†^ of Their Condition,^§^ by Race/Ethnicity — United States, National Health and Nutrition Examination Survey, 2011–2012 * Currently taking medication to lower blood pressure, based on affirmative responses to the following questions: “Because of your high blood pressure/hypertension, have you ever been told to take prescribed medicine?” and “Are you now following this advice to take prescribed medicine?” among those with hypertension. ^†^ Having measured systolic blood pressure <140 mm Hg and diastolic blood pressure <90 mm Hg among those with hypertension. ^§^ Measured systolic blood pressure of ≥140 mm Hg, diastolic blood pressure of ≥90 mm Hg, or currently taking medication to lower blood pressure. ^¶^ 95% confidence interval.

During 2011–2012, 75.6% of adults aged ≥18 years with hypertension were taking medication to lower their blood pressure, and 51.8% had their blood pressure under control. Non-Hispanic Asian adults with hypertension were less likely to be taking medication (65.2%) than were non-Hispanic black (77.4%) and non-Hispanic white (76.7%) adults with hypertension. No difference was observed in controlled hypertension among adults in the different race and Hispanic ethnicity groups.

**Source:** Nwankwo T, Yoon S, Burt V, Gu Q. Hypertension among adults in the United States: National Health and Nutrition Examination Survey, 2011–2012. NCHS data brief no. 133. Hyattsville, MD: US Department of Health and Human Services, CDC; 2013. Available at http://www.cdc.gov/nchs/data/databriefs/db133.htm.

**Reported by:** Tatiana Nwankwo, MS, bwt4@cdc.gov, 301-458-4553; Sarah Yoon, PhD; Steven M. Frenk, PhD.

